# Plant-Oil-Based Fibre Composites for Boat Hulls

**DOI:** 10.3390/ma15051699

**Published:** 2022-02-24

**Authors:** Agnieszka Dąbrowska

**Affiliations:** 1 Laboratory of Spectroscopy of Intermolecular Interactions, Faculty of Chemistry, University of Warsaw, Pasteura 1, 02-093 Warsaw, Poland; adabrowska@chem.uw.edu.pl; 2 Biological and Chemical Research Centre, University of Warsaw, Żwirki i Wigury 101 St., 02-089 Warsaw, Poland

**Keywords:** plant oil-based resins, natural fibres, composites, green materials, marine applications, hulls

## Abstract

Plant-oil-based fibre composites for boat hulls are an interesting and growing group of materials. Although many problems are to be tackled at different stages of their preparation, the green composites are already successfully commercialised. Within this paper, the most important chemical and physical characteristics of both natural fibres and sustainable resins are provided in the form of a comprehensive review. Finally, the complex issue of interactions filler–matrix is considered.

## 1. Introduction

**Figure 1:**
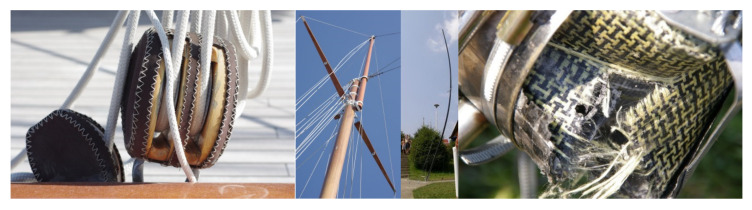
The classical wooden tackling versus the flexible composite mast is made from the polymer reinforced with aramid and carbon fibres.

As long as humans first encountered the blue ocean, its curiosity makes him search for the solutions to check what is behind the horizon. From the first rafts, canoes, kayaks, junks, via the variety of small fishing and sailing boats up to the golden era of tall ships and clippers, the natural materials were commonly used with the leading role of the wood. Rafts from balsa logs lashed together with inch and a quarter hemp ropes were used by indigenous people around the world and copied by Thor Heyerdahl for his famous Kon Tiki with only natural (bamboo reeds, banana leaves, mangrove wood, woven canvas, hemp yarn) elements. As the twentieth century brought the revolution of synthetic polymers synthesised at the global scale, the new type of materials has dominated the market. Plastics entered the shipping industry and significantly changed design engineering and production [1]. As cheap, versatile, easily formable and available, they soon became the ideal material for boat hulls and small equipment [2]. Moreover, their insufficient mechanical, thermal, or chemical resistance was soon overcome by reinforcing the polymer matrix with the different types of fillers. The discoveries proliferate with the following milestones: the introduction of fibreglass in the 1960s, sandwich constructions from the 1970s, 1980s with the aramid fibres (such as Kevlar), carbon fibres and vacuum-assisted methods of infusion (1990s) [3]. Although weaving of canvas sails from flax, cotton, hemp dates back to the beginning of sailing, the twentieth century brought the domination of synthetic materials and composites constructions (Figure 1). As a consequence, in the first decade of the twenty-first century, the epoxy and polyester resins reinforced by glass and carbon fibres were the most common materials used in maritime industry due to their durability in saline environments and matrices adhesive properties. However, the increasing problem of marine microplastics, the depletion of petroleum sources and growing environmental awareness changes the state-of-the-art. Currently, seeking green materials and sustainable solutions is observed. Therefore, the natural fibres are proposed instead of glass- or aramid ones and the polymer matrices in composites structures might be substituted with the biopolymers [4]. Within this chapter, a short and comprehensive overview of different approaches to the synthesis and applications of fibre-reinforced biomaterials is given. The hygrothermal ageing will also be discussed as a critical factor in terms of hulls production.

## 2. Composite Materials

**Figure 2:**
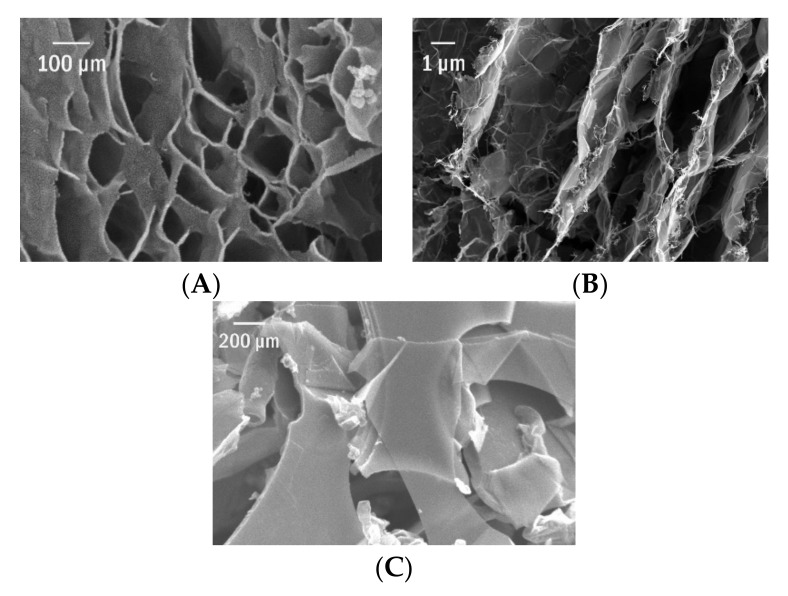
The SEM pictures of the variety of carbon-based nanocomposites fillers: (**A**) exfoliated carbon, (**B**) expanded graphite, (**C**) graphene flakes.

The twentieth century might be indisputably called the composite era. They can be classified accordingly to the type of matrix as metal, ceramics, or polymer ones. Together with the discovery and production growth of synthetic polymers, there has been a systematic study of their properties. Among the essential drawbacks, in terms of use as construction materials, one pointed out their insufficient mechanical resistance. The fibre reinforcement solves the problem. Two distinct phases joined together, to form a new material with the properties related to the contribution of both elements, are called the composites. The possibility of triggering the matrix by the optimal content of another substance added gave the new perspectives in material science. The traditional composites contained several dozen percentages of filler whose role was to enhance the polymer mechanical, thermal, chemical, electromagnetic properties. Regarding the maritime industry, the most popular renders to be the epoxy reinforced by carbon-, aramid-, or glass fibres. Composite materials have unquestionably dominated the market. In the boat-building industry, they overpassed the level of 5% and even 70% for units up to 50 m with the still-growing share. They have better resistance to corrosion, UV, seawater and the specific tensile strength (the strength-to-weight ratio) in comparison to the conventional materials together with enabling the complex shape creation. The first revolution was related to the nanomaterials in which the large surface to volume ratio enormously increases the interface region and adhesion matrix to filler. When they are used as fillers in the so-called nanocomposites (as one of the ingredients linear dimension do not exceed 100 nm), their content is much smaller (few % or even far below 1 phr) concurrently with the significant effect. That enables the saving of material and provides lightweight construction elements. The carbon nanotubes, graphene and different types of nanoparticles were considered (Figure 2). However, together with the increase of desirable properties, there is an escalation of leading fabrication and description problems. One may list the following:

- Anisotropy,
- Properties strongly depending on the fabrication method,
- Fillers agglomeration,
- Lack of homogeneity,
- Difficulties in the theoretical description and modelling (of for example the fatigue behaviour),
- Destructive phenomena at the interface,
- Self-heating and other specific effects [5],
- Void, oxygen and defects in the structure,
- Susceptibility to damage at binding and conjunctions where local stress is accumulated,
- Complex ageing behaviour.

**Figure 3:**
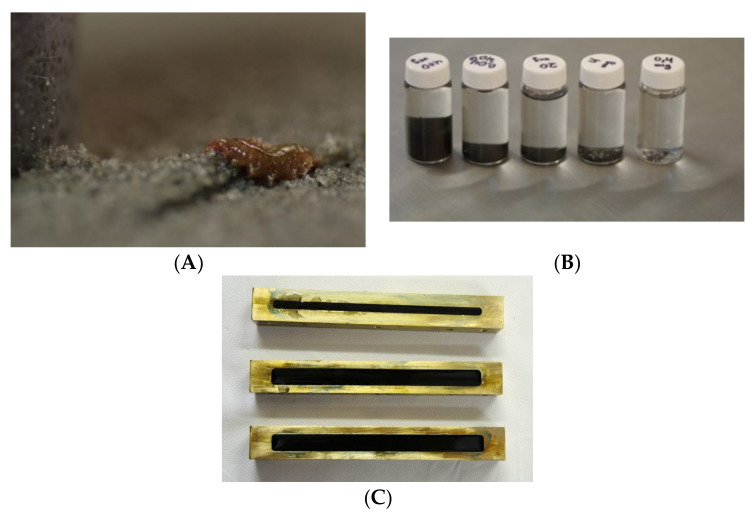
(**A**) *Hediste diversicolor* and (**B**) graphene flakes for ecotoxicology tests (as described in [8]) with different fillers that were also used for (**C**) nanocarbon modified composites.

The main concern is to secure the proper adhesion at the interface. The optimization of the synthesis parameters is crucial and is to be done separately for each combination filler/matrix. The choice of production method, mould shape, resin flow, temperature, type and content of filler, presence of other additives etc., will determine the properties of the final material. Another critical issue is related to ageing. As distinct from the classical and bulk materials, the degradation of the composite is subjected to a complex set of phenomena. Accelerated ageing studies revealed the deterioration at the interface matrix/filler and weakening of the adherence of components under high temperature as well as the decrease of elastic modulus for the epoxy resin. The delamination, matrix cracking, fibre fracture, fibre/matrix debonding are observed and studied. On the other hand, by definition, synthetic polymers are supposed to be one of the most durable. As a consequence of increasing production, insufficient recycling and the significant percentage of disposable items, a vast amount of plastic wastes is placed in the global ocean system. They are accumulated by prevailing winds and currents in the macroscale wires and further fragmented as exposed to the mechanical interaction, salt water and UV radiation. This problem was tackled also in my research as discussed from various perspectives in [6,7]. Finally, the microplastics (with a diameter less than 5 mm) concentration increases and poses a real threat to the biota, similarly to graphene nanofillers (Figure 3) as discussed in our previous study [8]. Moreover, during the degradation of composites, their components are released. Some of them are known to be strongly ecotoxic, such as the bisphenol A used in accessible epoxy materials. Considering all, one may seek a more environmentally friendly and sustainable alternative to polymer composites and plastics [9]. Furthermore, the development of biomimetics constitutes the new inspiration for the production of a biomaterial. Fillers and matrices are substituted by natural, comfortable scalable and recyclable materials. As they do not reach some of the unbeatable parameters of epoxy composites, the intermediate solutions are proposed and hybrid materials designed.

## 3. Natural Fibres as Sustainable Matrix Reinforcements

Although carbon and glass fibres are commonly used in the marine industry, natural materials seem to find their niche [10] successfully. For decades, petroleum-based composites were considered to be optimal in terms of durability, workability and cost. However, the environmental impact was not appropriately estimated. Taking into consideration the financial advantage of eliminating the CO_2_ footprint and the difficult recycling, the plant oil-based materials with natural fibres draw increasing attention despite some of their properties (e.g., significant moisture uptake, lower mechanical resistance). Due to the dominant role of fibres fillers in composites or nanocomposites and the growing environmental consciousness, natural fibres seem to be one of the most perspective materials. They are intrinsically polar and hydrophilic requiring additional treatments. Their use in the maritime industry has a long-lasting tradition, although the current research seems to be concentrated mainly on the complex sandwich constructions or structures with the prevalent content of the polymer matrix. The popular glass fibre/epoxy laminates are replaced by more sustainable natural fibres and biopolymers. However, complex theoretical modelling and complicated multi parametrical process optimization are still problems to be tackled. Moreover, the environmental costs related to the lands for agriculture and production wastes are not to be neglected.

### 3.1. Natural Fibres Overview and Classification

**Figure 4:**
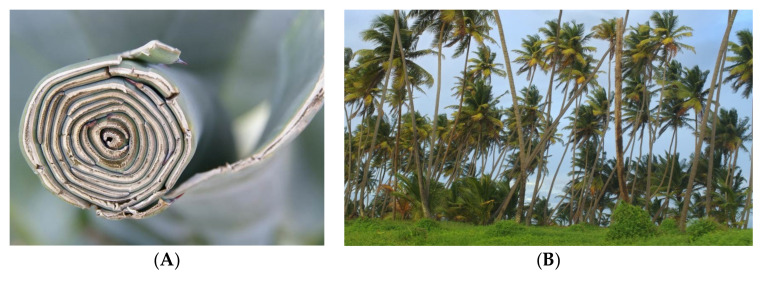
The example of the source for plant fibres: (**A**) *Agave Americana L.* and its structure, (**B**) palm forest in Trinidad Island.

In general, the natural fibres might be divided into two classes: those of plant and animal origin. Sometimes also the third group is named mineral-based fibres, such as asbestos (silicic rock). Their main advantages are low cost, low weight, excellent relative mechanical properties, biocompatibility, recyclability, abundance. However, high moisture sensitivity must be considered. Animal fibres are mainly the protein ones from wood or air of merino, crossbred, llama, horse, alpaca, mohair, vicuna, cashmere, camel, rabbit. So far, the silk fibre is for instance in sutures, but the plant-based materials (Figure 4) are much more used in the construction elements and maritime industry [11,12]. Among the most popular, obtained from fruits, seeds, bast or leaves, the following should be mentioned:

- Flax being easily woven,
- Hemp (Cannabia Sativa L.) [13],
- Kenaf (*Hibiscus cannabinus L.*) [14], which can reach the growing speed of 10 cm per day under optimum conditions,
- Jute (*Corchorus capsularis*) [15] with excellent temperature stability (up to 200 °C) and proper for woven materials [16],
- Sisal (*Agave sisalana*) with ~1000–1200 fibre bundles in a single (out of 200–250 for a plant) leaf,
- Coconut and coir with relatively good water (and saltwater) resistance,
- Pineapple leaf (*Anannus comosus*) [17],
- Henequen (*Agave fourcroydes*),
- Isora,
- Ferula,
- Okra (*Hibiscus esculentus*),
- Palf,
- Ramie (*Boehmeria nivea*),
- Abaca (Manila hemp),
- Bamboo (from *Bambusoideae* family) [18] with cellulose fibres in the lignin matrix, has a high strength to weight ratio, excellent temperature stability,
- Areca (*Areca catecu L.*) [19], originally from the Malaya Peninsula, the areca husk fibres are predominantly from hemicellulose,
- Banana (*Musaceae* family) [20] with 70 million metric tonnes of production per year,
- Cotton fibres with high impact strength but low stiffness,
- Bagasse,
- Miscanthus,
- Etc.

Floral fibres are composed of cellulose, hemicellulose, lignin, pectin and wax material. Proportions differ depending on the species. The composites filler is the cellulose (>80% wt in cotton, >70% in flax, palf, hemp, >60% in ramie, sisal, jute) which should be extracted without deterioration of its structure. Moreover, the carbon black from the bamboo stem, coconut shell or oil palm fibre bunch might be the efficient epoxy fillers [21].

### 3.2. Fibre Structure, Sources and Properties

Regarding the natural materials, one should focus on their general characteristics deeply considered in biomimetics. They are hierarchic, fractal, composed. This is an essential inspiration for contemporary engineering. Fibres are stiff due to the sufficiently high elastic modulus and tensile strength. Their mechanical properties increase with the lower microfibrillar angle (MFA), which is specific to the particular layer (one of three possible S1, S2, S3) in a given species. The lower the MFA, the higher the axial tensile modulus, as the helix is steep and fibrils closer to parallel to the long axis of a cell. The pitch angle determines how the fibril wind around the cell. The most favourable value is reported for the S2. In general, the structural fibres are the agglomerates of sclerenchyma cells sourced from the leaves of monocotyledon plants (sisal, abaca) or the bast of dicotyledons stems (hemp, flax). Mature sclerenchyma cells develop the rigid and thick secondary wall and die, leaving the hollow space (lumen) in place of the former cytoplasm. The presence of the hollow cavity enables significant weight reduction but is responsible for water retention. In each layer of the cell wall, the cellulose fibrils are coated with the amorphous layer of hemicellulose and pectin.

Cellulose, pectin and hemicellulose are the polysaccharides (chains of sugar molecules or sugar acids as in the case of pectin) but only the first one is desirable having much better mechanical properties due to the higher molecular weight (and so the tensile strength) and degree of crystallization (proportional to the stiffness) as well as the more regular, not branched structure, being composed uniformly of glucose. Cellulose forms several crystal structures, with four of them well studied. Cellulose I has a theoretical modulus of 134 GPa (higher than an aramid fibre), but once decomposed in a solvent, it will recrystallise as the more thermodynamically stable cellulose II with the modulus value of 90 GPa. Forms III and IV are not so frequent, and both return to the untreated structure when stored in a hot and humid environment. However, cellulose degrades when exposed to pyrolysis or the oxidizing agents due to the aldehydes and ketones formation out of the –OH groups and the subsequent elimination reactions causing the bond scission. Although being negligible below 170 °C, the change in crystallinity and degradation in alkaline media is to be kept in mind. Degradation of cellulose occurs by the hydrolysis of the glycosidic bond linking the glucose molecules in a chain. Hemicellulose, working as a fibres crosslinker, is easily extracted by warm water with diluted acid or base. It is mainly composed of highly branched polysaccharides (glucose, mannose, galactose, xylose, etc.). Pectin, containing galacturonic acid, binds cell walls and adjacent amorphous layers is capable of forming covalent and hydrogen bonds, ester linkages, ion bridges in the presence of calcium. Lignin fills space between cellulose fibrils making cells more rigid and reducing the permeability. It contains phenylpropyl groups connected by ether bonds. Being aromatic reacts under UV and in the presence of radicals [22]. The proportion between various components in selected fibres is presented in Table 1.

Fibres might be extracted directly from the purpose-grown crops (e.g., flax, hemp) or agricultural wastes (pineapple leaves, coconut husks). All cultivation and production steps will determine the properties of the final products whose average values are shown in Table 2. Furthermore, the part of the plant used influences its behaviour: seeds, fruits, leaves, stems, etc.). In particular, tensile properties and high specific strength are to be noted. Natural fibres also have a favourable aspect ratio and low density. The performance of cellulose ones depends strongly on the proportion between the lignin (lower moisture absorption) and hemicellulose (better thermal and biostability, lower UV degradation).

The highest stiffness of the natural fibres is one of the jute, ramie and flax. Flax fibres are known for their good damping properties.

### 3.3. Preparation and Pre-Treatment of Natural Fibres for Composites Fillers

Source and preparation of the fibres have a critical impact on their properties and performance as the reinforcement material [25]. The proper adhesion at the filler–matrix interphase is crucial for composite durability and resistance. It also enables efficient stress transfer. In the case of the natural fibres, their surface needs to be additionally modified due to insufficient wettability. Good adhesion to the matrix because of the developed surface is one of the fibres main advantages. Chemical modification aims to: obtain the filler–matrix interface that would be the wicking one, pure and to provide the proper adhesion at the interphase. The alteration of chemistry and morphology of the surface, the reduction of moisture retention and grafting of functional groups, can be obtained in numerous ways. Many are still unexplored but determining the performance of the fibre and so consist of the interesting space for advanced research. Pre-treatment also includes the protocol intended to remove unwanted compounds, such as lignin, pectin and hemicellulose. The aim is to extract the fibres without adversely altering their structures. A high aspect ratio is favourable.

The first step in order to obtain the valuable fillers from the raw plant material is to reduce the hydrophilicity and water retention of the fibres. First, the separation process is needed. It is essential to maintain the molecular weight of cellulose and its degree of crystallinity. Decortication, by hammermill or array of blades, serves to separate fibre from the shive (waste biomass). To facilitate the process, the selective weakening of binding agents is needed. The so-called retting has many variants, such as:

- Dew retting (pressed stalks are left on the ground to decay; the process is simple but difficult to control),
- Water retting (naturally occurring organisms degrade the plant),
- Controlled enzymatic retting (with chelators extracting calcium ions),
- Stand retting (which consists of spraying plants with a desiccant a few weeks before mean peak flowering; however, commonly used trimesium salt of the glyphosate might be carcinogenic),
- Retting by heat treatment (Duralin process; two-step stalks heating for the degradation of noncellulosic polymers hydrolysing to the lower molecular weight with the subsequent condensation on a fibre surface),
- Steam explosion (degradation of lignocellulose by the action of saturated steam at high temperature and under high pressure due to its rapid expansion).

As the studies on retting are mainly concentrated on hemp and flax, the more accurate approach to another type of fibres is to be elaborate.

Once separated, the refining of material is carried out by mercerization or bleaching. The first process is based on immersion in the alkaline bath (usually the aqueous sodium hydroxide), washing to the neutral pH and drying. As a collateral effect, the cellulose I may recrystallise to the weaker II form. The positive results are: removal of the hemicellulose, increased surface roughness, improved mechanical properties (flexural and tensile strength, the extension-to-break parameter, impact resistance). Bleaching removes the lignin, improve the dyeability, activate the surface for compatibilizers by the reduction of alcohols to aldehydes. Once performed mainly with sodium hypochlorite, it is now substituted by chlorine-free processes due to environmental concerns. The peroxides, ozone and strong acids are used. For instance, the coconut fibres treated with the combination of sodium hypochlorite and sodium hydroxide revealed reduced water retention and better performance [26]. On the other hand, in the material with low lignin content, blenching may cause cellulose degradation.

Furthermore, compatibilization is the process in which the reactive functional groups are introduced to form covalent and electrostatic bonds between the fibre and polymer. In general, the bonding occurs with the hydroxyl groups on cellulose. The most popular approaches are:

- Alkalization,
- The addition of silanes,
- Acetylation,
- Grafiting with the maleic anhydride.

Silanes in the form of R-Si-(OR’)_3_ are versatile materials as the R is a functional group compatible with the polymer matrix, whereas OR’ alkoxy group might be replaced by cellulose. Ethanol works as a deposition medium, and the reaction begins with the hydrolysis of the alkoxysilyl esters to silanol (Si-O-H). The acid or base works as a catalyst. Obtained silanols form hydrogen bonds with the fibre surface making it more hydrophobic or undergoes condensation reaction giving the silicate gels. For a covalent bonding formation, instead of just adsorption, a temperature above 100 °C is obligatory. It was shown that the silane with acrylate functionality could improve the flexural strength of composites (alfa grass/polyester). Hemp fibres have improved their tensile strength and Young modulus. It is also possible to graft silanes directly onto the matrix. In the agave fibres/polystyrene composites, the graft copolymerization of methacrylate with ceric ammonium nitrate as a catalyst was used. The same type of fibres, alkali-treated, reinforced epoxy composites.

Another simplified compatibilization approach in the case of plant fibres is the conversion of the hydroxyl groups of cellulose chains to esters. They are less hydrophilic and capable of forming interfacial bonding. Although the acetic anhydride is widespread, a variety of molecules might be used. It is also possible to graft the anhydride functionality onto the matrix directly as the activated carbons will form strong covalent bonds with hydroxyl groups of cellulose. For instance, the maleic anhydride grafts easily to polypropylene (forming the PP-g-MA) in the presence of radical initiators, whereas polylactic acid matrix works appropriately with the fillers acetyl esters. Hemp/PP-g-MA and jute/PP-g-MA have improved interfacial shear strength and flax/PP-g-MA hydrophobicity with respect to the not modified ones. Acetylation works not only as efficient compatibilization but also diminishes the equilibrium moisture content, which is crucial in the case of the maritime composites, disperse cellulose microfibrils by the elimination of alcohol groups and so the reduction of hydrogen bonding.

In 1938 the NO_2_ was used for the first time to oxidise cellulose selectively. Alkali and silicone treatment increased the flexural resistance of pineapple/nylon composites or in the coir fibres in the polypropylene matrix. The same positive effect was observed at coconut fibres (NaOH + silane) in a natural wheat gluten matrix. Finally, the variety of coupling agents (e.g., NaOH) were reported to modify the interactions at the interface filler/polymer resulting in the hike of mechanical parameters. It works well for example for the banana fibres into epoxy or the Palmyra palm-leaf stalk and jute fibres into unsaturated polyester matrix. The 3% maleic anhydride was an optimal coupling agent in the case of the ramie fibre/polylactic acid (PLA) composites and worked well also for the noil and scotched hemp fibres in polypropylene. Alkalization, acetylation and silane treatment were compared on hemp fibre for polyester. The 8% NaOH provide the best enhancement of flexural strength and modulus. To sum up, shortly, there is a variety of fibre chemical treatments used for the enhancement of composites properties. One may list the following tested processes (and chemicals used):

- Benzoylation (C_7_H_5_ClO),
- Cyclohexane modification (1:1 C_6_H_12_:C_2_H_5_OH),
- NaOH (5, 10 or 15%),
- Fluorocarbon,
- Silicon treatment,
- Isocyanate (CCl_4_ and C_32_H_64_O_4_S_n_),
- Peroxide (benzoyl or dicumyle 6% solution in acetone),
- Potassium permanganate (0.005% KMnO_4_ in acetone),
- Silane (5% in methanol),
- Sulfuric acid,
- Etherification (12 ml dodecane bromide into isopropanol solution),
- Maleic anhydride (20% solution in acetone),
- EDTA,
- Stearic acid, etc.

Diluted epoxy treatment is also an interesting one as, together with alkalization, proved to enhanced 40% longitudinal flexural strength, 60% stiffness and even 200% and 500% strength and stiffness in transverse fibres loading. One should note the 2% zein treatment as sustainable and efficient in the case of flax.

The physical treatment is also used, for instance, the plasma to improve flexural strength and modulus or irradiation with an electron beam. One may also use the autoclave thermal treatment, calendaring, sputtering, corona discharge or stretching. The mechanical activation (and aluminate coupling agent) helped in the fabrication of sugarcane bagasse fibres/PCV composites. Cured in an oven and microwave, hemp and kenaf fibres reinforced the epoxy matrix whereas oxygen plasma treatment (LF and RF systems) was successfully tested for jute in a high-density polyethylene matrix. The physical modifications aim to change the structural and surface properties as well as the hydrophobicity. For each material, the proper, different combination of treatments should be found and optimised. It can also provide the self-healing properties of the structure [27]. In some cases, animal sources are efficiently used. For instance, the chicken feather fibres were used in epoxy coatings [28].

Nowadays, numerous studies concentrate on agro-waste from industrial crops [29] as a source of fibres, and the prevention of their moisture uptake, which is a current bottleneck. The promising solution to this problem is based on the development of superhydrophobic surfaces [30]. They may resolve also the problem of the materials biofouling, microbial degradation and corrosion.

**Figure 5:**
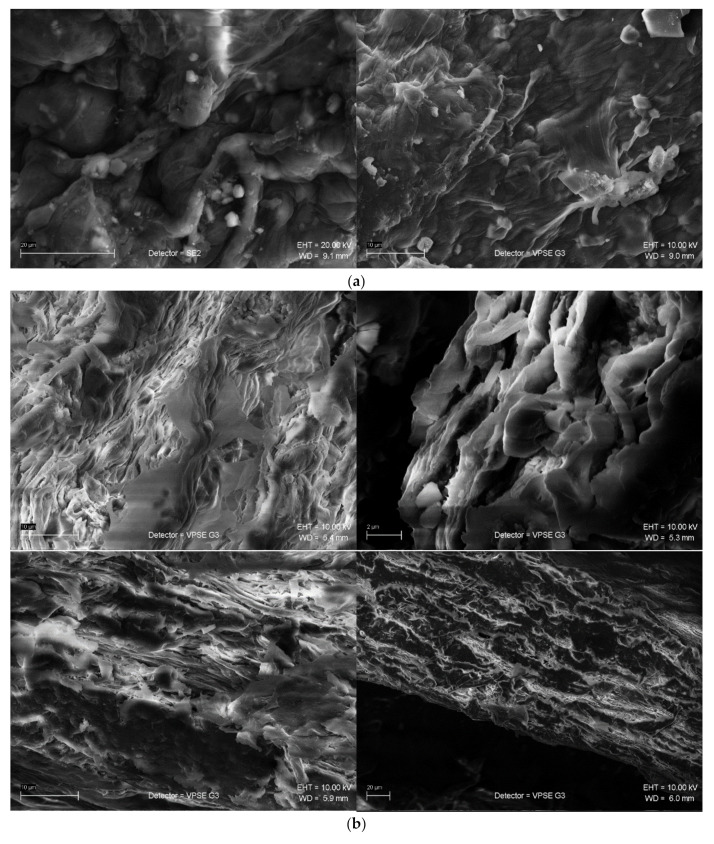

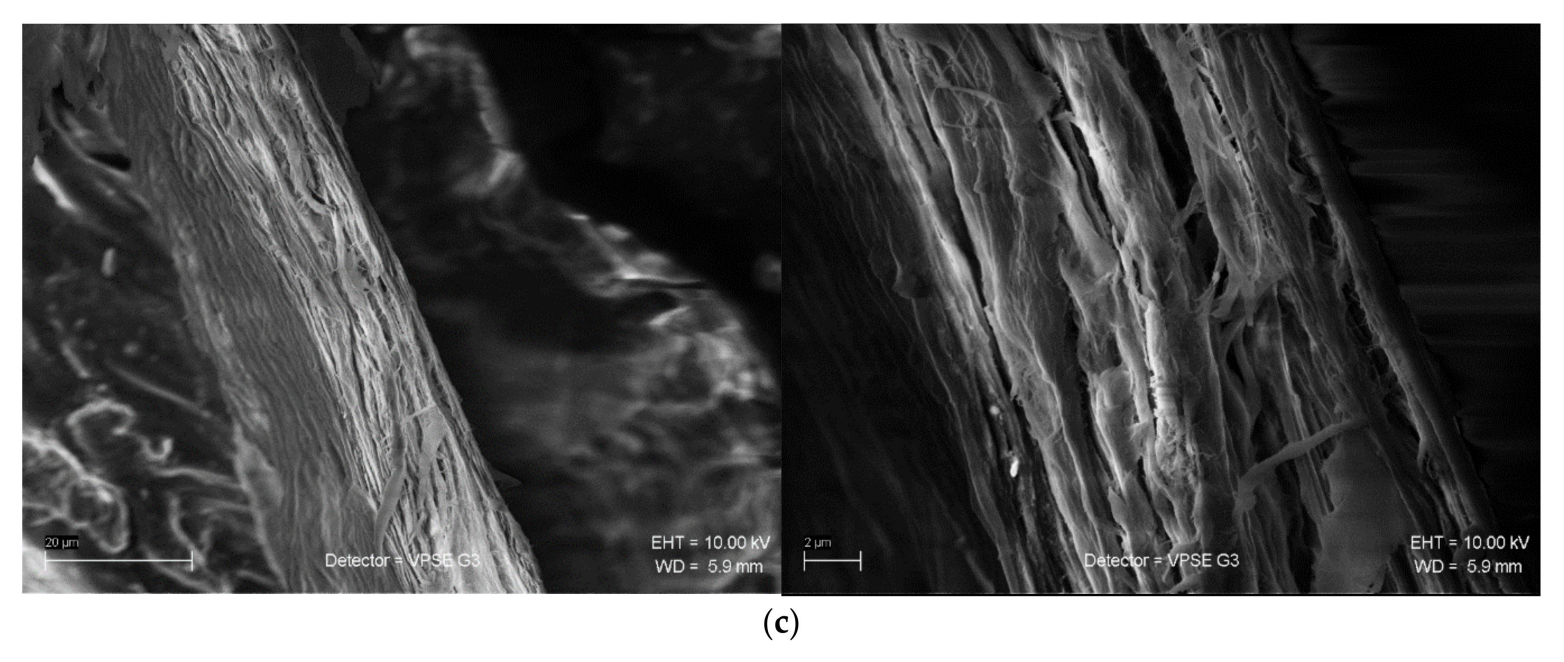
The SEM pictures of nanocellulose fibres from (**a**) garlic, (**b**) onion, (**c**) pineapple; @ samples thanks to the Poornima Vijayan P, Sree Narayana College for Women (affiliated to University of Kerala), Kollam 691001, Kerala, India, and SEM provided by Marcin Syczewski, University of Warsaw, Department of Geology, Warsaw, Poland.

Finally, one may point out that nanocellulose fibres [31], due to their high aspect ratio having the developed surface, will be the perfect fillers for composite materials. Changing the filler from macro to nanoscale may enhance the interactions at the interface. The roughness is correlated with the wettability of the fibre. Figure 5 presents an overview of the scanning electron microscope (SEM) pictures of nanocellulose fibres from selected natural sources. The energy-dispersive x-ray spectroscopy (EDS) analyses confirmed the traces of chemicals used for the purification and pre-treatment which is typical for that kind of materials.

## 4. Plant-Oil-Based Resins

The role of an already mentioned nanocellulose is crucial, and the material is extensively studied and used. It can be classified as cellulose nanocrystals, cellulose nanofibrils, bacterial cellulose [32]. However, to make it fully sustainable, bioresins are needed in composites production. Nowadays, the majority of classical composites modifications provide the natural fillers for the polymer matrix. Epoxy, polyurethane, polypropylene, polystyrene are among the most commonly used. However, to obtain a truly sustainable biomaterial, the green polymer should be introduced. That is the reason for the growing interest in natural, for instance, plant-oil based resins. Their main advantages include sustainability, versatility, low environmental impact and biocompatibility, variety, formability, functionalization. The low viscosity substances (<500 cP) are the most suitable due to the complex fabrication process of the composite structures. It is possible and recommended to obtain well-tried resins from natural materials such as vegetable oils [33] instead of petroleum. The click chemistry techniques are extensively studied. Bioepoxy resins can be obtained from rosin, furane, cardanol, eugenol or lignin. Cardanol-based phenolic resins from by-products in the cashew nut industry are successfully tested. Biobased and styrene-free vinyl esters are indicated as an important industrial solution in maritime applications [34]. Among the new generation of green resins, one can point out the poly furyl alcohol (PFA) obtained in a self-polymerization of the furfuryl alcohol under acidic conditions. Furfural can be obtained from biomass (corncobs, sugar cane bagasse or rice hulls) [35]. Tannins, herbal extracts, are considered to be a promising and sustainable source of bio-phenols and the substitute of synthetic ones [36]. It is not to be neglected, that the proper manufacturing, with the use of advanced techniques (such as the vacuum infusion), will determine the overall performance of composites [37]. Finally, the bioresins will increase the sustainability of fibre reinforces composites in what will be visible in their life cycle assessment and efficient mechanical, thermal or chemical recycling strategies [38].

### 4.1. General Overview of Materials

The standard epoxy resin is frequently used as the reference material when comparing the general performance. Although the majority of research is concerned with polylactic acid (PAL) or starch (amylose and amylopectin polysaccharides), nowadays other oils are tested. Among promising natural matrices for biopolymers, one can find:

- Polylactic acid (PLA) [39], which purified monomer (obtained from corn, rice, potatoes, sugar beet, agricultural waste) is polymerised in the presence of the suitable catalyst; current production is based on the fermentation (by fungi, yeast, bacteria),
- Poly(butylene succinate) (PBS) and poly(butylene-adipate-co-terephthalate) (PBAT) [40],
- Poly(ethylene succinate) (PES),
- Poly-L-lactic acid (PLLA),
- Polyesters containing polycaprolactone (PCL),
- Poly-3-hydroxybutyrate (PHB) from microbial fermentation,
- Poly-hydroxyvalerate (PHV),
- Poly(hydroxybutyrate-co-hydroxyvalerate) (PHBV),
- Poly-glycolide (PGA),
- Polyvinyl alcohol (PVA),
- Starch as an alternative to polystyrene,
- Cashew nut shell liquid (CNSL),
- Castor oil,
- Linseed oil (wit linolenic acids),
- Tung oil,
- Lingo phenolic resin,
- Soybean oil [41], acrylated epoxidized soybean oil (AESO),
- Cottonseed [42],
- Peanut oils,
- Oilseed radish,
- Cellulose acetate (CA) with a citrate plasticiser,
- Furan-based resins from furfural extracted from sugar cane bagasse, corn cobs, cereal by-products, wood,
- Others.

In general, thermosetting resins from vegetable oils are produced by grafting hydroxyl, acrylate and maleate moieties onto the fatty acid triglyceride. In the case of CNSL, the anarcardic acid is converted to cardanol (and some cardol), which is polymerised (free radical or condensation) in the presence of aldehydes. The presence of C=O bond enables the hydrolysis, and it is a reason for the water degradation of PLA. The sisal fibres-reinforced CNSL composites have a Young modulus ~8.8 GPa and a mean strength ~24 MPa. On the other hand, the popular biodegradable thermoplastic PBS is synthesised from the succinic acid and 1.4-butanediol. The critical parameter is the sufficient adhesion filler-matrix, but once obtained, the bio-composites properties provide a promising alternative to the traditional ones. For instance, one tested the jute fibre/PLA [43] enhanced the properties by alkali, peroxide, permanganate or silane pre-treatment. Moreover, hemp/PLA [44] after the silane and alkali treatment or alkali one alone [45] exhibited the improvement of mechanical parameters. The same effect was proved for kenaf/PLA [46] after alkalization and silane treatment or ramie fibres/polylactic acid (PLA) treated with alkali and silane. In another study [47], natural and human-made cellulose fibre-reinforced poly(lactic acid) (PLA) composites and their promising mechanical properties were tested. Jute yarns are co-mingled with thermoplastic PLA filaments derived from corn starch. The tensile properties (223 MPa) and flexural strength (254 MPa) of kenaf/PLA composites [48] increase linearly with fibre content (even 50%). Not only the polylactic acid derivatives are extensively studied. The by-product of the cashew nut industry has a high content of cardanol and is an interesting source for biocomposites. Cashew nut shell liquid composites were successfully reinforced by hemp [49] in a hand lay-up compression moulding. The research revealed that the optimal was 4 and 6% NaOH alkali pre-treatment (from 0.8–8% tested) to enhance the mechanical properties: Young’s modulus from 38 to 65 GPa and tensile strength from 591 to 1064 MPa, respectively. Caustic soda increases the number of hydroxyl groups, surface roughness and improves the fibre-resin adhesion. CNSL-formaldehyde resin with hemp fibre bundles seems to be a promising alternative for constructive elements. In other studies, the sunn emp/polyester (unidirectional, 40% vol.) composites had a higher specific stiffness than glass fibre (20% vol.) reinforced ones [50]. The natural fibres reinforcement worked well in hemp/ELO [51], non-woven jute fibres improved the strength and dumping of castor oil-based composites [52].

Finally, one is to note that although bio-thermoplastics were more extensively studied within the last years, the dominant role in hulls production is reserved for thermosets. They also provide the better impregnation of fibrous filaments, and the finer morphological structure is possible to be obtained. That is why they will be discussed in the following part of this paragraph.

### 4.2. Plant Oils as Raw Materials for Thermosetting Resins

Nowadays, plant oils are the most important raw material [53] for thermosetting resins production. The process requires various steps, and at each part of the synthesis, green substitutes are needed. For instance, the cashew nut liquid can be used as a green hardener for epoxy. It is important due to the increasing pollution by highly toxic and often carcinogenic curing agents. Bio-based epoxy resin hardeners for the maritime industry were proved [54] to provide materials of the same mechanical resistance and at the same time, lower moisture uptake. Phenalkamines and phenalkamides obtained from cardanol have good chemical and water resistance, adhesiveness and low curing temperature. Aromatic ring present in their structure is responsible for fire resistance, phenolic hydroxyl facilitates curing, whereas due to the long aliphatic chain resin can become hydrophobic and chain with amines improves the crosslinking density. As the adhesion at the matrix-filer interface is crucial, the different solutions are tested in order to improve it. For instance, the soybean oil derivative, PESO, is epoxidized SO and synthetised in a phosphoric acid ring-opening reaction. Plan oil enhances the hydrophobicity of composites by the presence of ester groups and unsaturated sites. In addition, the kenaf fibre is coated with poly(tannic acid) which enables the PESO to be an efficient cross-linker in the system [55].

In general, the plant oils are triglycerides, that is the triesters of glycerol with fatty acids. Their properties strongly depend on the molecular parameters and composition that varies due to the growing conditions, season, corps quality, etc. Parameters influencing the physical and chemical properties are mainly the following:

- Stereochemistry of the double bonds,
- Their location within the chain,
- The degree of unsaturation (0–7 carbon-carbon double bonds C=C),
- Length of fatty acids carbon chains (8–24),
- Presence of functionalities (e.g., epoxy in vernonia oil, hydroxyl in castor oil, furanoic or ester groups, etc.,),
- Possible co-monomers.

The iodine value is used to express the significant degree of unsaturation classifying the oils for drying (>130), semi-drying (90–130) or non-drying (<90). Drying oils are the most popular because of their ability of autoxidation and consequently the peroxide formation and radical polymerisation. The thermal or cationic polymerization with styrene or divinylbenzene might be used to obtain plastics. Cross-linked products are highly desired. Double bonds in vegetable oils are used in direct cationic or radical (co)polymerization with unsaturated monomers [56].

### 4.3. Phenolic Resins

The group of phenolic resins has a long tradition in the marine industry being the first used artificial material in many kinds of products because of their chemical, thermal and water resistance, good mechanical performance, dimensional stability, excellent ablative properties. They can be classified as novolacs (formaldehyde/phenol ratio 0.75–0.85) or resoles (substrates ratio > 1) on the ground of the acid or basic environment during synthesis. Hexamethylenetetramine can be used as a substitute for formaldehyde, and it is also the hardener of novolacs that have to be cured to obtain the cross-linked structure. Cashew nut shell liquid (CNSL) is a sustainable substitute of phenol. It contains four compounds, with a carbon C_15_ chain: cardanol, cardol, anacardic acid, 2-methylcarbol. Pure cardanol is obtained after the double vacuum distillation. Cardanol-formaldehyde resins are not only more environmentally friendly but have better flexibility and water resistance being less prone to weathering. The main drawback is their low tensile strength and porosity caused by the water release when cured. Cardanol can be chemically modified in a Mannich-like condensation to form the benzoxazine monomer. Another approach to substitute the phenol is the use of pyrogallol, which is the decarboxlade gallic acid from hydrolysed tara pods (*Caesalpinia spinosa*).

### 4.4. Epoxy Resins

The most common in maritime composites epoxy resins urgently needs more green substitutes. That is mainly due to the presence, in their structure or during processing, of the highly toxic and carcinogenic chemicals such as bisphenol A (2,2-bis(4′-hydroxyphenyl)propane) that is released directly to the environment during material ageing and degradation. More than 80% of world production is based on the reaction between the epichlorohydrin and bisphenol A giving its diglycidyl ether (DGEBA). Curing agents such as amines, amides (dicyandiamide DDA), anhydrides, acids, isocyanates are necessary to finish the synthesis of cross-linked material. The epoxy resins precursors contain at least one highly reactive epoxy functional group and might be obtained in more sustainable processes. Plant oils can be epoxidized but to carry out this process selectively and without ring-opening the new compounds are used. Castor oil, *Vernonia galamensis*, soybean oil are frequently epoxidized in the presence of a catalyst and the anhydrides as curing agents. Polymer networks obtained in that way would be potentially degradable in soil by the hydrolytic cleavage of glycerol ester bonds. Phosphorus-containing derivatives are favourable and drying linseed oils result in increasing hardness and T_g_. To enhance the mechanical properties, a two-step procedure is adopted. Soybean oil is transesterified by allyl alcohol and epoxidized in the presence of benzoyl peroxide before curing. Natural products also exhibit better water resistance and plasticizing effect than petroleum-driven polymers. The epoxidation by hydrogen peroxide along with catalyst and 1% of N-benzyl pyrazinium hexafuoroantimonate (BPH) curing agent is an efficient way to stable plastics [57]. With BPH the rapid conversion is observed at high temperatures (180 °C). Compounds with more double bonds have a higher oxygen per cent, selectivity and hydroxyl value. The epoxidation of plant oils is possible in one of the following ways by using:

- Percarboxylic acids
- Peroxides (organic or inorganic),
- Halohydrins,
- Molecular oxygen.

Among catalysts, one may find transition metal complexes or heterogenous titanium-silica. As epoxy resins are brittle, reactive rubber component such as acrylic rubber or hydroxyl-terminated butadiene is commonly used to enhance the toughness. Natural rubber, well-dispersed and bonded, might also be used as a reinforcing agent, compatibilizers, diluent [58,59].

### 4.5. Polyurethane Resins

Polyurethane resin is a macromolecule containing numerous urethane linkages (-NH-COO-). They are obtained by polyaddition of polyisocyanates and polyols. Sustainable materials contain renewable polyols and the aliphatic chains in vegetable oils work as soft amorphous segments. Currently, the effort is concerned to find the green substitute for isocyanate from petroleum that is highly toxic, causing, for instance, skin or eye irritation, respiratory problems. One approach is based on the soy polyurethane network without diisocyanates [60]. Another one uses vegetable amines and oil-based cyclic carbonates from CO_2_ and oxiranes in the presence of a catalyst (alkali metal halides, quaternary ammonium halides, polystyrene bound quaternary ammonium salts). Natural polyols are efficiently obtained from biopitches (oligomers from Eucalyptus tar after vacuum distillation), soy flours, castor oil [61]. The majority of production regards film, coatings, sealants, adhesives and foams. Plant oils are a promising source of sustainable polyols [62] after epoxidation, air oxidation, soft-hydrolysis, maleinization, hydroformylation, ozonolysis, transesterification (e.g., with glycerol), amidation. Unfortunately, the structure had defects in the network due to the presence of a double bond in a chain (so-called *dangling chains*). However, they may be chemically modified and work as plasticisers. Moreover, ozonolysis was an effective way to obtain the polyols, but as it requires the use of toxic solvent, it will not be regarded as a natural method. Soybean oil reacts with acetic acid and hydrogen peroxide in a one-pot process of epoxidation and ring-opening. Water acts as a cheap and natural nucleophilic reagent for ring-opening of epoxidized vegetable oils in a reaction with hydrogen peroxide and formic acid. The second generation of polyols from plant oil has a higher triol content, hydroxyl values and is prepared using ethyl acetate and zinc [63]. Together with aliphatic diisocyanates they form materials with high T_g_ and sufficiently good mechanical properties. Moreover, the introduction of aromatic or silicon compounds to the vegetable oils or their oxypropylation is a recommendable approach. Finally, the cardanol can be used as:

- Chemically modified by the addition of diethanol amine or by soft-hydrolysis,
- Reacting with glycerol monochlorohydrin.

Castor oil is suitable for polyurethanes production without any modification due to the inherent hydroxyl groups (~2.7 per glyceride). The even distribution is favourable for the formation of a uniformly cross-linked structure. Resins with aliphatic diisocyanate components have higher thermal stability than those with aromatic ones. For the solvent-free preparation of polyols from castor oil, epoxidized soybean oil (ESO) is efficiently used.

### 4.6. Polyester Resins

Polyesters are used for a variety of composites and play a significant role in boat building. The most common are the unsaturated polyesters from the polycondensation of a polyol (glycerol, ethylene glycol, trimethylopropane, neopentylglycol, pentaerythritol) and an acid anhydride (phtalaic or maleic) cured through radical or thermal processes with unsaturated comonomers (e.g., styrene); each of those components can be substituted by the green one. For instance, fatty acids or oils instead of polyacids, isosorbide from starch (rigid carbohydrate) as polyol or citric acid for improving coating properties. Cis-9, 10-epoxy-18-hydroxyocatdecanoic acid is a natural epoxidized monomer from the outer bark of birch or *Betula verrucosa* that can be polymerised in condensation reaction catalysed by lipase [64,65]. Interestingly, the birch trees can be a source of betulin also for another type of material. Acrylaed betulin added as a comonomer (5–10% wt) to the AESO yields coating material with improved abrasion resistance [66]. Hyper-branched poly(ester amides) were synthesised from N,N’-bis(2-hydroxyethyl)castor oil fatty amide and various anhydrides with diethanolamine. The large group of vinyl ester resins cross-linkers and reactive diluents are efficiently synthesised from plant-based feedstocks [67], and methacrylated ESO may substitute for example the toxic styrene (causing headache, fatigue, weakness, depression, central nervous or hearing dysfunction). Soybean oil is epoxidized and then acrylated to AESO.

### 4.7. Different Resins and Renewable Resources for Green Matrices Other than Plant Oils

Finally, not only plant oils are used as an alternative source of polymers. The proteins are regarded as a vast source for sustainable and biocompatible polymer materials, for example, soy seeds, milk casein, wheat or corn gluten. On the other hand, lignin is considered the most promising substitute for phenol in phenol-formaldehyde-based resins. It also works as a hard segment in epoxy resin or a source for unsaturated ester thermosets [68]. One should also mention the starch, inulin (the reserve polysaccharide of chicory *Cichorium intybus*), cork (after oxypropylation), chitosan and other carbohydrates, etc. Nowadays, natural materials are more often regarded not only as a more sustainable substitute of petroleum-based plastics but as a source of entirely new thermosetting materials. Heated and oxidised drying oils are a source of alkyd resins. Metathesis reactions (so-called ene reactions) serve as the initial activation of double bonds. It is crucial in the case of vinyl resins in which double bonds are not naturally conjugated and because of that need reactivation before the free radical or cationic polymerization. Suitable reactive vinyl moieties are introduced at the beginning. Isomerization serves for the conjugation of double bonds, and the acrylate or methacrylate groups are introduced to the chains. Only tung oil can be unmodified directly and fast polymerised at room temperature. Vinyl resins are ideal for self-healing elements and anticorrosion coatings.

One may conclude that it is much easier to find the green filler than a sustainable matrix at the same time fulfilling all harsh requirements of modern industry and the sea environment. It is still the edge of science where the majority of research efforts will focus on the proper use of the synergy effect at the interface. Moreover, the dynamic behaviour of green composites and their ageing is to be studied.

## 5. Plant-Oil-Based Fibre Composites Properties

Biocomposites are composite materials with at least one phase derived from natural origin. Regarding the biopolymers, one may focus on starch, cellulose or biodegradable polyesters (from microbiological production). Natural fibres are used as a matrix reinforcement. For instance, recycled paper is a cheap and sustainable source of cellulose fibres that may substantially enhance the mechanical resistance of the matrix. The main drawbacks are the generally high moisture absorption and the low impact strength. They are also not so stable at elevated temperatures. Among advantages, one may list the synergy effect of matrix-reinforcement interaction and the variety of formable products with versatile properties depending on the selection of components and the filler content. The proper filler dispersion in the polymer, sufficient wettability at the interface and defect-less structure are the critical parameters determining the general performance. Many properties depend also on the micro-fibrillary angle, which is between the fibre main axis and its angle in the structure and drops proportionally to the impact with the increasing cellulose content. The level of adhesion at the filler–matrix interface is described theoretically by the interfacial shear strength or measured in the fibre fragmentation, micro-bead or fibre pull-out tests. One may observe that the flexural properties will vary strongly depending on the fibre treatment [69], similarly to the fracture behaviour [70]. Those two parameters are crucial in further hulls performance. The overall performance of common epoxy composites was interestingly summarised by those authors [71,72].

In order to classify the materials and assess their physical and chemical properties, a lot of advanced techniques is used [73]. However, the methodology of measurements, as well as the synthesis methods, strongly bias the final results. That is why, they will be discussed in detail within this chapter [74]. Moreover, the hybrid composites are so far much more popular and consist of good reference material for general considerations about methods and properties. Due to that, some examples will be included. It is worth mentioning that the “hybrid” does not necessarily mean only green filler + synthetic matrix but may also note the positive synergy among different natural fibres. The better adhesion is due to the interlocking of fibres. For instance, the addition of up to 50% by weight banana into jute/epoxy resulted in decreasing moisture absorption and in increasing the mechanical and thermal properties [75]. A similar effect was confirmed for the sisal fibres in banana/epoxy [76]. Sisal is mainly added to form a hybrid filler with E-glass, cork, carbon, jute, banana, pineapple, kenaf, hemp or coir. The impact strength of sisal composites is lower after the fibre pre-treatment due to the enhanced adhesion at the interface. Hybridization of flax fibres with Lyocell ones diminishes favourably the water absorption [77].

Among the most popular natural fibres considered for maritime applications, one may find flax [78], sisal [79], kenaf and hemp.

### 5.1. Fabrication Techniques

As in the case of the synthetic composed materials, the chosen method of material production influences its final properties. Resin maintains the fibre position and transfers the load within its structure. Because of that, the proper orientation and dispersion to ensure the material homogeneity are crucial so precise process parameters control is needed, and their optimization is carefully considered. Processing conditions and techniques have a critical influence on material performance, modulus of elasticity, flexural stress and strain. For instance, the stiffness index is the function of the fibre volume and orientation factor. Among many fabrication methods [80,81,82,83] of biopolymers one may point out:

- Film stacking,
- Film blowing,
- Vacuum-assisted resin infusion,
- Resin transfer moulding (RTM),
- Vacuum-assisted resin transfer moulding (VARTM),
- Hand lay-up,
- Compression moulding,
- Filament winding,
- Injection moulding,
- Pultrusion (mainly for long and uniform cross-section parts),
- Pre-forming method (PF),
- Pre-preg sheet method (PS),
- Direct extrusion,
- Compounding,
- Extrusion,
- Commingling, etc.

One can find in the literature also: twin-screw extruder and hydraulic press, tubular braiding technique, brabender internal mixer and compression moulding, hydraulic press using film stacking technic, powder impregnation through compression moulding and extrusion followed by injection moulding, woodturner and die, melt mixing (plastomill) and compression moulding, solvent mixing followed by the hot press, shear K-mixer and injection moulding, droplet method, haake rheomix and injection moulding. All of the listed approaches have their specific modifications depending on the type of resin and, without an exaggeration, almost each experimental and production set-up might be regarded as unique. On the one hand, that may be treated as the advantage of providing the enormous versatility of materials. On the other hand, reproducibility and large-scale optimization are challenging. The ease of fabrication is one of the most important parameters when seeking new material. Furthermore, each method has its particular advantages and drawbacks [84] and fibre type, length, orientation, diameter, surface modifications and content are to be considered.

In popular injection moulding, the solid pellets are melted, passed through the sprue nozzle and put into the mould cavities with filler. The fibre length is critical, as too long are under high attrition during the process and too short may work as a matrix defect rather than reinforcement. Other problems are related to the volume expansion, residual stress because of rapid cooling, water molecules trapped inside micro-fibrils, warpage and shrinkage of the product. The following parameters should be optimised: melt temperature, injection and screw speeds, mould temperature, injection pressure, mould shape and size. For PLA the temperature window is 150–210 °C, but under the same conditions, the semicrystalline polymer would have a higher shear viscosity than an amorphous one.

Compression moulding is the combination of hot-press and autoclave processes: thermoplastic prepregs are placed in a mould and treated with the programmed heat-pressure cycle with curing at the end. One should avoid excessive pressure to eliminate the risk of fractures and air bubbles, introducing defects in the product. Moreover, the temperature gradient is needed to maintain the same environment in all volumes and avoid overheating on the surface or minor heat transfer to the core. Tensile properties of composites decrease with the decreasing flow velocity during fabrication. Due to the stress relaxation, and deformation during cooling, the slight excess of material should be placed in a mould. The uneven fibre distribution is one of the main obstacles, but additional fabrications steps enable the proper distribution of the filler. The process is simple and widely used.

In the variety of liquid composite moulding processes, one may distinguish RTM (resin transfer moulding), VARTM (vacuum-assisted resin transfer moulding), S-RIM (structural reaction injection moulding), CIRTM (co-injection resin transfer moulding), etc. The aim of preparing new methods is to enhance their efficiency, yield, reproducibility and products quality by controlled injection of the liquid to the bed of stationary preforms. The air bubbles or turbulent flow are to be eliminated. Usually, the major variables are the temperature, permeability, viscosity, gate and vent configuration and location, pressure, velocity and flow.

Spray impregnation followed by compression moulding is an efficient approach to the preparation of soybean oil-based composites (from acrylated ESO, methacrylated soybean oil, methacrylic or acetic anhydride modified soybean oil) with various fibres mats (flax, hemp, flax-polyester) [85].

Solution blending, in particular adequate for thin films, was used for kenaf/chitosan nanocomposites [86].

Pultrusion is a method to obtain high-quality profiles adequate for industrial needs and meet the requirements of structural materials. Research confirmed that material obtain by this method is dimensionally stable and resistant to ageing maintaining its mechanical properties [87]. Pultrusion is especially adequate for forming a uniform and long cross-sections. Its main advantage is the continuous process and good mechanical properties of products. One of the highest Charpy Impact Energy (115 kJ m^−2^) was reported for the unidirectional pultruded flax/PP composites.

The stacking technique produces composites by heating pellets under pressure and forming isotropic laminates. In contrast, just one step is required for vacuum bagging, which is the up-graded (providing fewer defects) hand lay-up technology. The uniform dispersion is possible, for instance, in the extrusion where the filler is mixed with melt polymer and mixture conveyed through the die to form extrudates.

To conclude, the proper method depends on the application of the product and should be optimized individually.

### 5.2. Characterization Methods and Selected Properties

One may point out the insufficient mechanical parameters being significantly lower for natural than carbon or glass fibre. However, the cellulose materials have promised the relative values (concerning the density) of mechanical resistance and Young modulus. Moreover, the electrical resistance and acoustic insulating properties are important advantages. The important for composites and mostly evaluated parameters are the following:

- Tensile, flexural, impact, inter-laminar, hardness properties (mechanical features),
- Water absorption and resistance,
- Thermal properties,
- Tribological properties.

For the theoretical modelling, commonly used are the rule of mixture:

E = E_f_V_f_ + E_m_V_m_

and Halpin-Tsai model:

$$E\;=\;E_{m}(\frac{1+\xi \chi V_{f}}{1-\chi V_{f}}),\;\mathrm{where}:\;\chi \;=\frac{E_{f}+E_{m}}{E_{f}+\xi E_{m}}$$

where: E—modulus of composite, E_f_—modulus of fibre, E_m_—modulus of composite, V_f_—volume fraction of fibre, V_m_—volume fraction of matrix, ξ—Halpin-Tsai parameter that serves to adjust the modulus value between the maximum for the fibre and the minimum for the matrix.

This is not the only theoretical approach to the mechanical properties of biocomposites. One should mention, among many others, the inverse rule of the mixture or Shear-lag theory.

The Tsai-Pagano model is efficiently used for the prediction of Young’s modulus in composites with randomly distributed fillers.

The Fourier-transform infrared spectroscopy (FTIR), x-ray diffraction (XRD), dynamical mechanical analysis (DMA), thermogravimetric analysis (TGA) and scanning electron microscopy (SEM) characterisation methods are the most popular and useful to determine the chemistry and physics of composite materials. Regarding the mechanical performance, one may carry on the standard approach [88,89,90,91], such as the three-point bending test. The DMA analysis provides information about T_g_ and thermal stability, crosslinking in the resin network, viscoelastic behaviour, the degree of molecular mobility. It is possible to calculate the Young’s, storage and loss modulus, damping factor or estimate the heat deflection temperature. For instance, flexural properties, such as load, strength, modulus, deflection at the break, are carefully assessed in the case of the structural materials. The flexural modulus is defined as follows:

$$E_{f}=\frac{L^{3}m}{4bh^{3}}$$

where: E_f_—flexural modulus of elasticity, L—support span, b—width of the beam, h—thickness of the beam, m—slope.

There is a relationship between the fibre length, orientation and content and the flexural strength with the optimum depending on the type of material. According to the study on kenaf/PLA composites [92], the elastic moduli, tensile and flexural strength increased linearly with fibre content (up to 50%). A similar result was obtained for the kenaf/polypropylene composites [4]. The flexural strength of nearly 200 MPa (193.75) was obtained for the banana/E-glass-reinforced polyester [93]. The epoxy matrix was reinforced with birch, palm and eucalyptus fibres [94] obtaining the values of 29, 53, 42, 24, 45, 28 MPa for the tensile stress, 58, 83, 68, 58, 79, 92 MPa for bending stress and 0.105, 0.130, 0.124 J for impact energy, respectively. Hybrid laminates are increasingly popular. In the case of jute/epoxy, the introduction of an additional e-glass thin outer layer improved the tensile, bending and impact properties [95]. Comparing the impact strength of fibres, one may conclude that the coir is better than jute and kenaf. However, it has the worst other mechanical properties. The banana-flax in epoxy resin composites [96] were prepared by manual hand layup method. Laminates of flax and banana have excellent impact resistance as the cracks cannot propagate between different structures. Investigations of hybrid phenol-formaldehyde-based composites reinforced with glass revealed that the oil palm fibres hybridised with filler resulted in improved impact strength [97]. Similar enhancement is observed for the sisal fibres and silica nanoparticles, whose presence diminished the porosity. To effectively enhance the mechanical properties, the high impact-resistant fibre should be added to the outer layer and the tensile strength ones to the inner part [98]. To improve further the impact resistance, the fibre coating (silicate, isocyanate) is recommended, e.g., sodium lauryl sulphate for banana/kenaf [99]. It was shown that the inter-laminar shear strength and fracture toughness of the hybrid composites were higher [100] and among different fibres, kenaf specific modulus and strength are better than those of sisal, coir or E-glass [101]. The hardness seems to be the only mechanical parameter linearly decreasing with an increase of fibre content. Additionally, one can carry out the Rockwell hardness test, wear test, rheological experiments, fracture toughness-compact tension, static compression testing to better characterise the particular material.

Tribological properties of materials are temperature-dependent and related to the friction and wear that usually cause the dissipation of energy and deterioration of properties. Micro-cracks and debonding are predominantly responsible factors. Rice straw and rice husk dust have improved the tribological properties of composites [102] and sugar cane was even better in terms of wear than a glass fibre [103].

To evaluate the thermal properties, thermo-gravimetry (TG) and differential scanning calorimetry (DSC) are useful. The proper crystallization degree, transition and melting temperatures are crucial. Moreover, in this case, the filler pre-treatment can enhance the final properties. In particular, at TGA, the untreated kenaf/epoxy lost weight earlier than other samples. Fibres improved the pure matrix. In another study, the thermal conductivity and diffusivity declined with banana fibre loading in polypropylene. Moreover, fibres treated with higher NaOH concentration showed better thermal and physical properties [104]. Positive influence has also an agar on the sugar palm starch composites [105]. The e-glass/soybean oil-based polyurethane composites have mechanical and thermal properties comparable with the petrochemical materials [106]. Thermal diffusivity, conductivity and fire reaction properties are also important parameters.

Among other methods for physical and chemical characterization scanning electron microscopy (SEM) is particularly useful to show the fraction mechanisms, cracking, interface morphology, material cross-section or surface roughness. The distribution of cells or empty spaces corresponding to the lumen in fibre is observed. Moreover, the void propagation after the impact tests is possible to track. furthermore, the TEM, optical, confocal or polarization microscopies are essential for the study of morphology. In addition, infrared spectroscopy (FTIR) can show the covalent bonding and functional groups what is important during surface modification or chemical pre-treatment. The wear and ageing of materials can also be described. Three peaks at 1725 cm^−1^, 1646.9 cm^−1^ and 1376.6 cm^−1^ disappeared indicating the carbonyl, double bond C=C and methyl group dissociation. The peak at 1625 cm^−1^ is absent in the alkalized kenaf (because of the lack of hemicellulose), whereas the 1512 cm^−1^ is attributed to the presence of aromatic rings in lignin and the absorption band at 1359 cm^−1^ is due to the C-H bending in hemicellulose and lignin. Other spectra techniques, such as Rama nor NMR, are also a valuable source of information about the structure and configuration of the macromolecules.

The XRD shows the crystallinity index and size of coherent domains in the material. Interestingly, the fractal analysis can be used for a detailed description of the interface matrix–filler. It is not only a qualitative but also a quantitative approach to numerical surface classification [107].

There are numerous advanced theoretical models of polymer and composites structures. Regarding the laminates fabrication, Darcy’s Law might be useful to characterise the permeability that is by the flow behaviour of the resin in contact with the fibres layer. The fluid flow through porous media can be described by the following parameters:

$$\bar{u}=\frac{K}{\mu }\frac{\mathrm{dP}}{\mathrm{dx}}$$

where: $\bar{u}$—the average velocity, K—permeability parameter, μ—viscosity of the injected fluid, P—pressure, x—flow direction, and:

$$K_{\mathrm{uns}}={\left(\frac{Q}{A}\right)}^{2\;}\frac{\mu }{\left(1-V_{f}\right)}\frac{1}{\mathrm{dPdt}}$$

where: K_uns_—unsaturated permeability, A—cross-sectional area of the mould cavity, V_f_—fibre volume fraction in the cavity, μ—viscosity of the injected fluid, dP/dt—the pressure change rate.

Finally, porosity is one of the main reasons for different mechanical behaviours observed for apparently the same composites. It strongly depends on the filler content, length, wettability, processing techniques. By optimization of them, the quality of products increases. The void content in the composite can be described as follows (ASTM D 2734-91):

$$V_{p}=100-M_{d}\left[\frac{r}{d_{r}}-\frac{g}{d_{g}}\right]$$

where: V_p_—void content in the composite [%], M_d_—measured composite density, r—resin content [wt%], d_r_—resin density, d_g_—fibre density, g—fibre content [wt%]. The cross-linked densities of materials can be estimated owing to the Flory–Stockmayer theory and are directly related to their T_g_ as in Fox and Loshaek model. The T_g_ and tensile strength of polymers were predicted using the vector percolation theory. The critical stress (σ_c_) breaking the thermoplastic network is described with the formula:

$$\sigma_{c}=\sigma_{0}E\vartheta_{c}{\left(P-P_{c}\right)}^{1/2}$$

where: σ_0_—the constant proportional to the energy of disentangling or break bonds, E—tensile modulus, ϑ_c_—the critical entanglement density, P—a level of perfection of the network, P_c_—percolation threshold, and the strength of triglyceride polymers is estimated as follows:

$$\sigma_{c}={\left(\sigma_{0}E\vartheta \right)}^{1/2}$$

The tensile strength improves with increasing functionality level (exponentially with the number of acrylates).

Those approaches were efficient for the description of triglyceride-acrylates prepared from various oils and copolymerised with styrene [108].

### 5.3. Hygrothermal Ageing

The hygrothermal ageing of the plant oil-based composites, one of their main drawback, is intensively studied [109]. The moisture uptake has a significant effect on the flexural properties and failure modes. The testing standard is ASTM D570. Being hydrophobic, plant fibres absorb a considerable amount of moisture. Hemp fibres retain more than 40% of their dry volume in water. They have to be properly dried before the composites fabrication as the presence of water deteriorates adhesion filler–matrix [110]. Flax fibre, according to the empirical rule, can absorb more than 10% of moisture in less than 1 h at room temperature and 90% humidity. Moreover, droplets may be the reason for voids formation weakening the material, reducing its tensile strength and elastic modulus. Water absorption influences the shape, debonding and loss of strength.

To reduce moisture absorption, one may try the following approaches:

- Laminating an inner layer of NFC with an outer layer of conventional synthetic fibre composite,
- Improving the compatibility of the matrix with the plant fibre,
- Chemically treat or the resin coating or the plant fibres.

For instance, the alkali-treated kenaf reduced the moisture uptake from 6.38 to 3.85% [111] due to the lower content of hemicellulose and the minimal voids. Furthermore, the hybridization with e-glass considerably reduces water adsorption. The specific impact of salt water on the constructive materials was studied in many research [112,113,114,115]. Moreover, the fabrication techniques are selected to provide the optimal performance at sea [116]. One can also observe the significant difference between various resins. For instance, linseed oil, castor oil and epoxy were compared. After the 46 weeks in the water at 40 °C, the worst performance obtained for the glass/linseed oil composites was probably due to the changes in failure modes. Its flexural modulus was reduced by nearly 60% affected by 26 weeks of ageing. On the other hand, the glass/castor oil samples exhibited the lowest reduction of flexural strength (21% after 22 weeks of ageing) being comparable with the epoxy resin composites. Plant oil-based resins do not reach the moisture equilibrium even after 46 weeks. The moisture uptake is linear at the first phase and reaches equilibrium after a few weeks (~6) of immersion. The long-term performance (>20 weeks) is more complex than an instant effect with a saturation of ~2–4%. Flax/PLLA biocomposites tensile strength has reduced by 70% due to the hydrolysis of the matrix, structural changes, swelling at the interface [117]. In one of the research, the 13–31% drop of the sisal/polypropylene composites strength was observed for samples immersed into the liquid. Observing the reaction of kenaf/unsaturated polyester composites [118] immersed in the different solutions, one may conclude that the moisture absorption is the slowest in the salt water due to the ions blocking the diffusion paths. For the studied material, the absorption rate slows after ~20 days and reaches saturation after ~260 days. In general, the following processes are mainly responsible for the deterioration of the material [119]: polymer plasticizing, reduction of the glass transition temperature, matrix cracking, delamination, fibre damage, changes at the interface (which fails to transmit the load from matrix to fibre), extensive delamination, higher voidage, the introduction of defects [120,121,122,123,124]. Among the several models of humidity ageing one should name the following:

- Plasticization of the matrix,
- Concentration gradient causing the differential swelling,
- Embrittlement due to the degradation of the macromolecular skeleton caused by hydrolysis,
- Osmotic cracking,
- Hydrothermal shock and change of the water state,
- Damage at the matrix-filler interface.

As a result, the mechanical properties and dimensional stability deteriorate. Moisture penetrates the composite structure:

- By diffusion into the gaps between polymer chains,
- Due to the capillary transport into the gaps and flaws caused by the insufficient wettability and incomplete impregnation,
- Involving the micro-cracks of the matrix.

The percentage of water absorption is described by the following formula:

$$\mathrm{abs}\;\%=\frac{m_{2}-m_{1}}{m_{1}}$$

where: m_1_ and m_2_ are the mass of dry and wet samples. The diffusion coefficient characterised the ability of water molecules to move among the polymer segments. The product of diffusion and sorption is the permeability coefficient.

In one of the recent works [125], the hygrothermal ageing in salt and freshwater and its effect on mechanical properties were studied for the laminates (short fibre/woven). Although they were made not from natural materials (fibreglass and unsaturated polyester), some considerations about process mechanisms are universal. The effect strongly depends on the time, medium and temperature. Moisture uptake is possible because of the presence of voids at the filler–matrix interface and the free volume of the polymer. Besides, some resins (like epoxy) are prone to water in which presence occurs their hydrolysis or plasticization. Kinetics of the water sorption is obtained by monitoring the change in time mass of the immersed specimen. The initial slope of the curve is proportional to the diffusion coefficient (greater for distillate than salt water), whereas the plateau corresponds to the saturation effect. Comparing the data from 48-h tests at 40 °C and 60 °C, one should note with increasing temperature occurs a decrease in ultimate stress, yield stress, Young’s modulus and the increase of elastic and failure strains. The breaking strain improved after a month of ageing (at 60 °C)

Finally, UV radiation is an important ageing factor to be considered during materials design, especially for applications at high seas.

## 6. Selected Hybrid Constructions and Their Applications

Although entirely biocomposites (matrix and filler of the natural origin) are intensive studies but still at the stage of research, the hybrid materials already have their numerous applications [126,127]. For instance, polyester and polypropylene are reinforced with flax or hemp. Sisal is added mainly to the high or low-density polyethylene (HDPE, LDPE), high impact polystyrene (HIPS). The synergy effect is used to enhance the interactions at the matrix–filler interface. Many different hybrid approaches are extensively studied [128,129,130,131]. However, joining cause local stress accumulation, and in the case of the hybrid materials, the problem is much more complicated than with the classical composites. The entirely green composites are implemented in automotive [132,133], aircraft industry, sports equipment (fishing rods from CelluComp Ltd. with nanocellulose from root vegetables), electronics, in furniture, bridges, roofing and infrastructure [134], or everyday use items and toys [135], off-shore energy platforms, noise barriers, impact-resistant covers, wind turbine blades [136]. For example, the Isonat is an insulation material made from hem and polyester (15%) fibres. Coconut fibres in natural rubber (latex) were used at a large scale in the automotive industry. Already in 1996 epoxy with jute was used in E-class and in 1999 the inner door panel in S-class Mercedes-Benz contained 35% of elastomer and 65% of a blend of sisal, hemp and flax. In 2000 Audi presented the A2 car with the door trim panel of polyurethane with flax/sisal reinforcement whereas Toyota developed the bioplastics from sugarcane. Moreover, the kenaf/glass fibre epoxy materials are popular for the car bumper beam [137] and sisal/rosell hybrid for the inner elements and accessories [138,139]. Moreover, biomedical applications, in the case of protein fibres, are possible. In the search for reliable and effective structures, naval architects and boat builders are seeking materials on the edge of science. Composites for fishing vessels are frequently made in a contact moulding process of laminate fabrication. Regarding the maritime industry [140], among other already tested applications one may find for instance: the canoe comprising flax skins (FLaxland), surfboards and boats from the EcoComp UV-L resin (Sustainable Composites and Movevirgo Ltd.), Mini Transat 6.5 with 50%: 50% flax: carbon fibre in a resin (Huntsman Advanced Materials), canoe, surfboards, kite surfers, yachts with Lineo’s FlaxPly and FlaxPeg, flax-epoxy catamaran (by Aero Skimmer design), surfboards with hemp or bamboo [141]. In a series of research, the entirely natural matrices were reinforced by various types of carbon nanotubes preparing the following composites with enhanced mechanical, thermal and chemical properties: MWCNTs/castor-oil based polyurethane [142], silane-modified-MWCNTs/soy-castor oil-based polyurethanes [143], MWCNTs/hyper-branched polyurethane from castor oil [144], MWCNTs/soy-based polyurethane foam [145]. Also, GO (graphene oxide), and graphene may be used. It was easier to disperse the larger than smaller MWCNTs in a soybean-oil polyurethane matrix due to the lower specific surface and so the weaker van der Waals interactions [146].

Finally, a variety of research is currently carried out in that domain. For instance, one of the leading scientific groups [147] works within the project called the SeaBioComp, which aims to develop bio-based thermoplastics appropriate for applications in the marine environment (e.g., boats, energy farms, buoys, pontoons, etc.) and with limited impact on it.

## 7. Conclusions and Future Perspectives

The increasing demand for environmentally friendly materials draws attention to the application of green composites in the maritime industry. That sector includes next to ship hulls mentioned here, also propeller blades, tidal turbine blades or components of off-shore platforms and wind farms. Plastics are no longer considered cheap and unexcelled as long as the proper maintenance, recycling and all life cycle of the material are to be considered. Biocomposites are already the promising alternative for glass fibre/epoxy and other conventional composites. As their use is still limited due to the significant moisture uptake, hybrid materials are designed. Although promising, they should be regarded as the intermediate stage before the application of biopolymers with entirely plant-oil based resins and natural fillers in dominant branches of industry, including the maritime one. The recently grown companies extensively promote green solutions [148]. Among the advantages of plant fibres, for instance flax, one may list the following: density (being ~1.5 g cm^−1^ it makes it about 40% lighter than dominant glass), capability to absorb vibrations and noise, making the workplace safer and cleaner and environment friendly.

The author recommends considering nanofibers of plant origin as a next step towards the materials of a new generation. They are supposed to be the superposition of desirable properties of natural substances and nanomaterials. Adequately prepared, by grinding, cutting, etc., might increase the surface of filler–matrix interface, provide a significant reduction of percolation threshold, enhance mechanical properties. Finally, the tendency of polymers employed in rigging is regarded as currently dominating the market [149]. Substitution of Dyneema (ultra-high molecular weight polyethylene) shackles with biopolymers seems to be a matter of time. Past years [150,151] have brought an outbreak in versatility, resistance, durability, production or self-healing [152] of biomaterials. For instance, the epoxy resins that can be polymerised underwater open new perspectives for materials fabrication and maintenance. The coir fibres [153] are popular and often added to PLA matrix. Those types of matrices are one of the most popular biopolymers. One can consider also including recycled materials in hybrid constructions. Moreover, the eco-friendly materials from wastes of leather industry or other animal sources (e.g., cow hair) had their successful preliminary tests in composites with unsaturated polyesters [154]. Fibre-reinforced composites (FRC) will remain the leader of the market, however, already at this stage, the source and structure of the fibre itself are open for the introduction of natural materials, also in a nanoscale.

As they did in the past, the natural materials will lead us through the oceans towards a more sustainable future.

## Figures and Tables

**Table 1:** The chemical composition and moisture content in the selected popular plant fibres.

Fibres	Cellulose [%]	Hemicellulose [%]	Lignin [%]	Pectin [%]	Moisture [%]
Flax	71	18.6–20.6	2.2	2.3	5–10
Hemp	70–74	17.9–22.4	3.7–5.7	0.9	2–6.2
Jute	61–71.5	13.6–20.4	12–13	0.2	8
Kenaf	45–57	21.5	8–13	3–5	
Sisal	66–78	10–14	10–14	10	10–22
Banana	63–64	10	5		10–12

**Table 2:** Some of the properties of the selected natural fibres [23,24].

Fibre	Elastic Modulus [GPa]	% Elongation	Tensile Strength [MPa]	Young Modulus [GPa]	Density [g/mL]
Hemp	70	1.6	550–900	30–80	1.48
Flax	60–80	1.2–3.2	345–2000	15–80	1.45
Sisal	38	2–14	300–700	10–30	1.33–1.5
Kenaf		1.6	200–900	22–60	1.3
Jute		1.5–1.8	300–700	13–55	1.3
Ramie		2–3	400–938	61–128	1.5
Bamboo		1.4	190–600	21–50	0.6–0.9
Banana		1–3.5	161–780	7.6–9.4	0.72–0.88
Coconut			150–180	4–6	1.2
Pineapple		0.8–1	126.6	4.4	1.07
Coir		15–40	175	4–6	1.2
E glass	73	3.4	2400	72	2.55
Kevlar 29	70.5		2920–4100	130	1.44
Nylon 66	3.5		85		1.14
1080 steel	207		2550		7.9
Carbon fibre		1.3–1.8	~4000	235	1.4

## Data Availability

Data sharing is not applicable to this article.
